# Supplementary material for: Selective inhibition in CA3: A mechanism for stable pattern completion through heterosynaptic plasticity
Source: PLoS Comput Biol. 2025 Jul 7;21(7):e1013267. doi: 10.1371/journal.pcbi.1013267 (PMC12244581; doi:10.1371/journal.pcbi.1013267)
Supplement: Attachment — Submitted filename: Response to Reviewers.pdf [file pcbi.1013267.s002.pdf]

Response to Reviewers

PCOMPBIOL-D-24-01892
Selective inhibition in CA3: A mechanism for stable pattern completion through heterosynaptic plasticity

Dr. Bush and Dr. Berry,

Thank you for coordinating the review of our manuscript. We have revised the paper extensively, adding new empirical citations, shortening the Results and Discussions, inserting testable predictions, and comparing our work with prior models. All page and line numbers below refer to the clean revised manuscript ("Manuscript.pdf").

Reviewer #1.

1. **Lack of Empirical Justification for Theta-Modulated Trisynaptic Pathway.**

   Response.

   Section 4.1 Biological plausibility of the model (p 16, lines 346-355) has been rewritten to show that CA3/CA1 firing and pathway gating are modulated by theta phase (Douchamps et al., 2013; Siegle et al., 2014; Lurie et al., 2022; Ku et al., 2024). We link these findings to our routing rule in which mossy fiber drive dominates at the trough, whereas perforant-path and recurrent collateral drive dominate at the peak (Malezieux et al., 2020; Villarreal et al., 2007). While the data align with our assumptions, direct phase-locked measurements across the full trisynaptic loop have not yet been reported; the first sentence of final paragraph of 4.1 now highlights this empirical gap.

2. **On/Off Switching of CA3 Projections Lacks Justification.**

   Response.

   Immediately following the text above (p 16, lines 356-362) we now add a limitations paragraph that places the model's binary 'on/off' rule in biological context. It

acknowledges that CA3 afferent gain is controlled by multiple, partially overlapping mechanisms—cholinergic modulation, interneuron-specific disinhibition, and short-term synaptic plasticity—and therefore our phase-locked silencing of PP or Rc should be viewed as a first-order simplification. We cite in-vivo work showing graded, not binary, modulation of mossy fiber and Rc efficacy across the theta cycle (Zucca et al., 2017; Ahmadi et al., 2025). These additions clarify the intent and limits of our modelling assumptions.

3. **Lack of Testable Predictions & Experimental Implications**

Response.

New Section 4.3 *Experimental predictions* (pp 17-18, lines 380-426) proposes three falsifiable experiments:

1. Memory-specific plasticity onto CA3 interneurons. A TRAP / in-vivo Ca^{2+} imaging / ex-vivo recording pipeline should reveal heterosynaptic potentiation of mossy-fibre input after learning.

2. Necessity of inhibitory-engram cells for competitive recall. Intersectional opto-/chemogenetic silencing (TRAP × PV-Cre / GAD2-Cre) is predicted to abolish winner-take-all retrieval.

3. Inhibition level during encoding sets engram size and recall bias. Transiently reducing CA3 inhibition while learning should let an enlarged engram dominate later recall unless E→I heterosynaptic plasticity is intact.

Each experiment is paired with the required tools (two-photon imaging, whole-cell physiology, chemogenetics) and behavioural read-outs, satisfying the request for actionable predictions.

4. **Connections to Prior Work Are Missing.**

Response.

We have added a new Discussion subsection, 4.4 Previous models of the hippocampus (page 18, lines 427-453). This section now reviews three lines of work that use full-circuit, theta-phase models to improve on simple Hebbian learning. We first summarise

the error-driven framework introduced by Ketz et al. (2013), in which alternating encoding and recall phases deliver a powerful error signal. We then describe Schapiro et al. (2017), whose model couples Hebbian and error-driven rules to let the monosynaptic entorhinal-CA1 pathway learn statistical regularities while the trisynaptic pathway memorises individual episodes. Finally, we discuss the Theremin model of Zheng et al. (2022), which pushes the error signal into CA3 by treating the delayed dentate-gyrus pattern as a teaching signal for CA3 recurrent and EC to CA3 synapses—a mechanism they link to heterosynaptic plasticity.

After laying out this background, the subsection explains how our study diverges from these predecessors. Where the earlier models update synapses by comparing activity across successive theta half-cycles, our model keeps encoding and retrieval strictly segregated and instead explores how heterosynaptic plasticity onto engram-specific CA3 inhibitory neurons can stabilise memories by suppressing competing traces during recall. By shifting the focus from weight-based error correction to inhibitory gating, the new text makes clear where we build on prior theories and where we offer a distinct advance, thereby addressing the reviewer's concern.

5. **Revise the manuscript for clarity by eliminating redundancy and improving text coherence.**

   Response.

   We performed a line-by-line edit to tighten language and remove repetition; all changes are visible in Revised Manuscript with Track Changes.pdf.

Reviewer #2.

1. **In Fig. 2B, authors show that interneurons exhibit a broad range of storage capacity. Is that correlated to firing rate or burstiness of these neurons? Are higher mean firing interneurons involved in more assemblies?**

   Response.

   Capacity is driven chiefly by the number of mossy fiber contacts each CA3 interneuron receives. Cells with a denser DG input fire more often during the encoding phase, which makes them more likely to cross the plasticity threshold and become linked to

additional assemblies. Thus the broad range in Fig. 2B reflects heterogeneity in DG input, not momentary high firing within individual theta cycles.

2. **In Fig. 4A, why is the activity of hilus layer excitatory neurons higher than inhibitory neurons? It is clear the hilus (inh) basically reflects sup EC inputs, but it is not clear why that would drive higher activity in hilus (exc) neurons.**

Response.

We have clarified this point in subsection 3.3 Feedforward dentate gyrus (p 9, lines 161–163). The new sentence explains that, although hilus excitatory neurons are subject to local inhibition, they also receive a stronger direct drive from superficial EC than the hilus interneurons do. This dominant excitatory input outweighs the local inhibitory tone, so the net firing rate of hilus excitatory cells remains higher and provides the sustained excitation needed to keep GCL inhibitory neurons active. Including this explicit rationale resolves the apparent discrepancy noted by the reviewer.

3. **Authors state that they adjusted peak conductance and did not modify synaptic weights to maintain connection probability. However, connection probability might still remain statistically similar in case of enabling weight updates and infact could shed more light on the underlying mechanisms. A point to include in discussion perhaps. Also, please cite Song et a., 2000. Nature Neuro., original paper that used the above conductance-based approach.**

Response.

Section 5.4 now explains why we changed only the peak conductance of existing synapses and left the binary connection matrix untouched. The STDP rule we use contains a potentiation term but no LTD branch, and the CA3 recurrent circuit is silenced during learning. Under those two conditions new contacts could be created but none removed, so allowing binary weights to change would push the overall connection probability steadily upward, contrary to the balanced creation/elimination assumed by Song et al. (2000, Nat. Neurosci.). Scaling conductance preserves the intended probability while still letting synaptic strength adapt, which is sufficient for the questions we address. The rationale and the citation to Song et al. have been added to the text.

4. **The authors compare selective inhibition vs. global inhibition In Fig. 8-10. But it seems like the results of selective inhibition include selective inhibition + global inhibition (line 172-176). It might also help compare results with a model with selective inhibition alone if possible. Diving more into Fig. 8H results might also help parse it out.**

Response.

We realised the wording in the original submission was ambiguous, because global inhibition is present in every simulation; the experimental contrast adds or removes selective (engram-specific) inhibition on top of that baseline. To make this explicit, we replaced the phrase "selective inhibition vs. global inhibition" throughout the Results with "with selective inhibition vs. without selective inhibition," making it clear that global (population-wide) inhibition is the default in both conditions.

Also, we added a paragraph in §3.2 (p 7, ll 141-149) that now explains the temporal interplay: Selective inhibition acts first, biasing the cue toward the target engram without fully silencing other assemblies; global inhibition, driven by the ensuing recurrent excitation, then suppresses residual activity indiscriminately.

We did not simulate a condition in which global inhibition was completely removed. In our framework global, population-wide inhibition arises automatically from baseline E to I synaptic weights (see Materials & Methods, Fig. 5A3) and represents ubiquitous feed-back inhibition reported in vivo. Nevertheless, we expect that a selective-only network would still bias activity toward the target engram, but the noise level would rise because nothing would suppress residual activity in non-tagged assemblies after the initial bias.

5. **It would be good to extend more on Fig. 5D results. Is that something the authors expect to see in empirical work?**

Response.

Yes. Empirical work already shows that weakening CA3 or amygdala interneurons enlarges the ensemble that forms, whereas normal inhibition keeps it compact; we therefore expect that progressively increasing dentate-gyrus activation in intact tissue will plateau at a nearly constant CA3 engram size, while the same protocol delivered

during transient interneuron suppression will let ensemble size grow with input strength. These two outcomes—size-invariant recruitment under normal inhibition and scalable recruitment when inhibition is weakened—provide a straightforward experimental test of the mechanism illustrated in Fig. 5D.

6. **The authors list only benefits of selective inhibition, but are there any downsides of it?**

Response.

In our simulations we did not detect any performance cost relative to the baseline with global inhibition alone. However, we acknowledge a theoretical limitation: selective inhibition is most efficient when each inhibitory interneuron responds preferentially to a small set of excitatory assemblies. In the extreme, if every memory required its own "grandmother-cell" interneuron, the network's memory capacity would become constrained by the size of the inhibitory population—an arrangement that is neither biologically plausible nor efficient. In our model each inhibitory cell can participate in several engrams, mitigating this issue, but we agree that excessive stimulus-selectivity could reduce capacity and remains a point for future investigation.

7. **Line 501: Why such a high rate (50 Hz) was required is not explained. Similarly high values are in Line 248-249, etc. It might be better to state that some firing rates are biologically unrealistic in the Results sections also to help readers. On the same lines, in the similarity analysis, neurons with firing > 25 Hz are considered. Do the results change significantly if neurons with firing rate say 5-10 Hz are included.**

Response.

1. Methods & Materials, Section 5.1 now includes a brief justification (p 18, lines 477-481): we frame the 50 Hz tonic input as a stand-in for gamma-rhythm excitation (≈30-100 Hz) that ensures sufficient entorhinal drive for stable engram formation. The text emphasizes that this is a modelling convenience rather than a physiological claim.
2. Results, Section 3.2 now contains an explicit caveat (p 7, lines 212-214) noting that the 80-120 Hz trains are model-driven winner-take-all activity; in vivo CA3 pyramidal cells fire ≈0.3-5 Hz with brief ~200 Hz bursts. We explain that the elevated sustained rate sharpens competition and has no bearing on the qualitative conclusions.
3. The original 25 Hz cutoff corresponds to three spikes within the 120 ms theta phase

that defines each retrieval cycle. In preliminary runs we found that background activity—either from random noise or from losing engrams before a winner emerges—can reach two to three spikes per cycle, so a 25 Hz threshold cleanly excludes this transient noise while remaining well below the sustained 80–120 Hz volley produced by the winning assembly. We have now inserted a clarifying sentence in Methods, Section 5.5 stating explicitly that "this sustained 25 Hz activity exceeds the ~0.3–5 Hz normally reported for CA3 pyramidal neurons in vivo and is used here solely as an analysis criterion." To address the reviewer's concern, we re-ran every analysis using a lower, biologically tighter cutoff of 8.3 Hz (one spike per 120 ms, within the suggested 5–10 Hz range); all qualitative conclusions were unchanged.

8. **It would also be good at look at changes in the I to E connectivity even though the focus is E to I connections.**

Response.

In the model all I to E synapses were kept fixed. We made this choice deliberately for isolating the mechanism of interest. Our central claim concerns how heterosynaptic potentiation of E to I synapses can form inhibitory engrams that bias recall. Allowing I to E plasticity at the same time would introduce an additional learning loop and make it hard to attribute effects to a single mechanism. We agree that exploring I to E changes is an important next step, but it lies outside the scope of the present study.

9. **In Fig. 2D, I would say percentage overlap rather than overlap ratio, as ratio suggests a value between 0 and 1.**

Response.

We agree with the reviewer's suggestion. Throughout the manuscript every instance of "overlap ratio" has been replaced with "percentage overlap," including the y-axis label and caption of Fig. 8H, the corresponding passages in the Results, and the Materials and Methods. No other content was affected.

10. **I would recommend the authors cite works of Dr. Marlene Bartos and Dr. Attila Losonczy's group (Rolotti et al., 2011, Neuron; Haimueller et al., 2024, Nature Comms) on inhibitory plasticity.**

Response.

Both references have been added at the two points where we discuss inhibitory plasticity at Introduction and Discussion.

We thank the reviewers and editors once again for their constructive feedback and for considering our revised manuscript.
