# Supplementary material for: Selective inhibition in CA3: A mechanism for stable pattern completion through heterosynaptic plasticity
Source: PLoS Comput Biol. 2025 Jul 7;21(7):e1013267. doi: 10.1371/journal.pcbi.1013267 (PMC12244581; doi:10.1371/journal.pcbi.1013267)
Supplement: Attachment — Submitted filename: plosreview.docx [file pcbi.1013267.s001.docx]

Summary

The authors used a spiking neural network model to examine engram formation in CA3. Rather than global inhibition, which is traditionally used, the authors proposed selective inhibition induced by heterosynaptic plasticity as an alternative. They showed that selective inhibition leads to sparse distributed coding in CA3, better retrieval performance, and enhances stability of pattern completion in different conditions (overlapping engrams, number of competing engrams for a single cue, different size engrams, etc.). Their model aligns well with the pattern completion and separation experimental findings for different hippocampal sub-regions. The paper presents a significant advance and is of sufficient interest to merit publication. I have some concerns that are outlined below.

1. In Fig. 2B, authors show that interneurons exhibit a broad range of storage capacity. Is that correlated to firing rate or burstiness of these neurons? Are higher mean firing interneurons involved in more assemblies?
2. In Fig. 4A, why is the activity of hilus layer excitatory neurons higher than inhibitory neurons? It is clear the hilus (inh) basically reflects sup EC inputs, but it is not clear why that would drive higher activity in hilus (exc) neurons.
3. Authors state that they adjusted peak conductance and did not modify synaptic weights to maintain connection probability. However, connection probability might still remain statistically similar in case of enabling weight updates and infact could shed more light on the underlying mechanisms. A point to include in discussion perhaps. Also, please cite Song et a., 2000. Nature Neuro., original paper that used the above conductance-based approach.
4. The authors compare selective inhibition vs. global inhibition In Fig. 8-10. But it seems like the results of selective inhibition include selective inhibition + global inhibition (line 172-176). It might also help compare results with a model with selective inhibition alone if possible. Diving more into Fig. 8H results might also help parse it out.
5. It would be good to extend more on Fig. 5D results. Is that something the authors expect to see in empirical work?
6. The authors list only benefits of selective inhibition, but are there any downsides of it?
7. Line 501: Why such a high rate (50 Hz) was required is not explained. Similarly high values are in Line 248-249, etc. It might be better to state that some firing rates are biologically unrealistic in the Results sections also to help readers.
   On the same lines, in the similarity analysis, neurons with firing > 25 Hz are considered. Do the results change significantly if neurons with firing rate say 5-10 Hz are included
8. It would also be good at look at changes in the I to E connectivity even though the focus is E to I connections.
9. In Fig. 2D, I would say percentage overlap rather than overlap ratio, as ratio suggests a value between 0 and 1.
10. I would recommend the authors cite works of Dr. Marlene Bartos and Dr. Attila Losonczy’s group (Rolotti et al., 2011, Neuron; Haimueller et al., 2024, Nature Comms) on inhibitory plasticity.
